# Supplementary material for: High-Intensity Training for Knee Osteoarthritis: A Narrative Review
Source: Sports (Basel). 2023 Apr 20;11(4):91. doi: 10.3390/sports11040091 (PMC10141118; doi:10.3390/sports11040091)
Supplement: Supplementary file 1 [file sports-11-00091-s001.zip › sports-2331658-supplementary.pdf]

Table S1. Table of the excluded studies with reasons for exclusion and references

#	Author	Year	Title	reason for exclusion
1	S. Aciksoz, A. Akyuz and S. Tunay	2017	The effect of self-administered superficial local hot and cold application methods on pain, functional status and quality of life in primary knee osteoarthritis patients	no high intensity training
2	A. I. Adhama, M. O. Akindele and A. A. Ibrahim	2021	Effects of variable frequencies of kinesthesia, balance and agility exercise program in adults with knee osteoarthritis: study protocol for a randomized controlled trial	no high intensity training
3	M. A. Ahmad, A. H. MS and A. Yusof	2022	Effects of low-level and high-intensity laser therapy as adjunctive to rehabilitation exercise on pain, stiffness and function in knee osteoarthritis: a systematic review and meta-analysis	no high intensity training
4	A. Alonazi, S. Hasan, S. Anwer, A. Jamal, S. Parvez, F. A. S. Alfaiz and H. Li	2021	Efficacy of Electromyographic-Biofeedback Supplementation Training with Patellar Taping on Quadriceps Strengthening in Patellofemoral Pain Syndrome among Young Adult Male Athletes	no high intensity training
5	A. Angelova and E. M. Ilieva	2016	Effectiveness of High Intensity Laser Therapy for Reduction of Pain in Knee Osteoarthritis	no high intensity training
6	N. M. Brisson, A. A. Gatti, P. W. Stratford and M. R. Maly	2018	Self-efficacy, pain, and quadriceps capacity at baseline predict changes in mobility performance over 2 years in women with knee osteoarthritis	no high intensity training
7	J. E. Broderick, F. J. Keefe, P. Bruckenthal, D. U. Junghaenel, S. Schneider, J. E. Schwartz, A. T. Kaell, D. S. Caldwell, D. McKee, S. Reed and E. Gould	2014	Nurse practitioners can effectively deliver pain coping skills training to osteoarthritis patients with chronic pain: A randomized, controlled trial	no high intensity training
8	J. Byra and K. Czernicki	2020	The effectiveness of virtual reality rehabilitation in patients with knee and hip osteoarthritis	no high intensity training
9	S. Cakir, S. Hepguler, C. Ozturk, M. Korkmaz, B. Isleten and F. C. Atamaz	2014	Efficacy of Therapeutic Ultrasound for the Management of Knee Osteoarthritis	no high intensity training
10	M. B. Corkery, C. P. Hensley, M. K. Danilovich, V. S. Davila and K. K. Chui	2021	Power Training in Older Adults With Knee Osteoarthritis	no high intensity training
11	B. L. De Kock, J. Van der Gragt, H. F. Van der Molen, P. Kuijer and N. Zipfel	2023	What Personal and Work-Related Characteristics of Dutch Construction Workers With Knee Osteoarthritis Are Associated With Future Work Ability?	no high intensity training
12	N. J. Desale and S. Anandh	2020	Effectiveness of progressive resistance drills and dynamic balance training on functional performance among the patients with osteoarthritis of knee	no high intensity training
13	I. Dobrydnjov, C. Anderberg, C. Olsson, O. Shapurova, K. Angel and S. Bergman	2011	Intraarticular vs. extraarticular ropivacaine infusion following high-dose local infiltration analgesia after total knee arthroplasty: a randomized double-blind study	no high intensity training
14	N. Dogan, H. Yilmaz, B. Ince and S. Akcay	2022	Is Kinesio Taping Effective for Knee Osteoarthritis? Randomised, Controlled, Double-blind Study	no high intensity training
15	R. B. Ferraz, B. Gualano, R. Rodrigues, C. O. Kurimori, R. Fuller, F. R. Lima, A. L. De Sá-Pinto and H. Roschel	2018	Benefits of Resistance Training with Blood Flow Restriction in Knee Osteoarthritis	no high intensity training
16	N. Foroughi, R. M. Smith, A. K. Lange, M. K. Baker, M. A. F. Singh and B. Vanwanseele	2011	Lower limb muscle strengthening does not change frontal plane moments in women with knee osteoarthritis: A randomized controlled trial	no high intensity training
17	S. Heywood, J. McClelland, P. Geigle, A. Rahmann, E. Villalta, B. Mentiplay and R. Clark	2019	Force during functional exercises on land and in water in older adults with and without knee osteoarthritis: Implications for rehabilitation	no high intensity training
18	K. K. Ho, L. C. Lau, W. W. Chau, Q. Poon, K. Y. Chung and R. M. Wong	2021	End-stage knee osteoarthritis with and without sarcopenia and the effect of knee arthroplasty - a prospective cohort study	no high intensity training
19	N. Johns, J. Naylor, D. McKenzie, B. Brady and J. Olver	2022	Is inpatient rehabilitation a predictor of a lower incidence of persistent knee pain 3-months following total knee replacement? A retrospective, observational study	no high intensity training
20	Z. Ju, X. Guo, X. Jiang, X. Wang, S. Liu, J. He, H. Cui and K. Wang	2015	Electroacupuncture with different current intensities to treat knee osteoarthritis: a single-blinded controlled study	no high intensity training

21	A. R. Kheshie, M. S. Alayat and M. M. Ali	2014	High-intensity versus low-level laser therapy in the treatment of patients with knee osteoarthritis: a randomized controlled trial	no high intensity training
22	G. J. Kim, J. Choi, S. Lee, C. Jeon and K. Lee	2016	The effects of high intensity laser therapy on pain and function in patients with knee osteoarthritis	no high intensity training
23	D. Kobsar, S. T. Osis, B. A. Hettinga and R. Ferber	2015	Gait biomechanics and patient-reported function as predictors of response to a hip strengthening exercise intervention in patients with knee osteoarthritis	no high intensity training
24	T. Kurien, L. Arendt-Nielsen, K. K. Petersen, T. Graven-Nielsen and B. E. Scammell	2018	Preoperative Neuropathic Pain-like Symptoms and Central Pain Mechanisms in Knee Osteoarthritis Predicts Poor Outcome 6 Months After Total Knee Replacement Surgery	no high intensity training
25	A. K. Lange, B. Vanwanseele, N. Foroughi, M. K. Baker, R. Shnier, R. M. Smith and M. A. F. Singh	2009	Resistive Exercise for Arthritic Cartilage Health (REACH): A randomized double-blind, sham-exercise controlled trial	no high intensity training
26	C. J. Liu and N. K. Latham	2009	Progressive resistance strength training for improving physical function in older adults	no high intensity training
27	M. S. Miller, D. M. Callahan, T. W. Tourville, J. R. Slauterbeck, A. Kaplan, B. R. Fiske, P. D. Savage, P. A. Ades, B. D. Beynnon and M. J. Toth	2017	Moderate-intensity resistance exercise alters skeletal muscle molecular and cellular structure and function in inactive older adults with knee osteoarthritis	no high intensity training
28	N. Mirmaroofi, A. Ghahramanian, M. Behshid, F. Jabbarzadeh, T. C. Onyeka, M. Asghari-Jafarabadi and J. Ganjpour-Sales	2019	Relationship between self-efficacy and pain control in Iranian women with advanced knee osteoarthritis	no high intensity training
29	M. Mostafa, H. A. Hamada, A. M. Kadry, S. S. Zahran and N. A. Helmy	2022	Effect of High-Power Laser Therapy Versus Shock Wave Therapy on Pain and Function in Knee Osteoarthritis Patients: A Randomized Controlled Trial	no high intensity training
30	A. Nazari, A. Moezy, P. Nejati and A. Mazaherinezhad	2019	Efficacy of high-intensity laser therapy in comparison with conventional physiotherapy and exercise therapy on pain and function of patients with knee osteoarthritis: a randomized controlled trial with 12-week follow up	no high intensity training
31	B. E. Oiestad, E. Quinn, D. White, F. Roemer, A. Guermazi, M. Nevitt, N. A. Segal, C. E. Lewis and D. T. Felson	2015	No Association between Daily Walking and Knee Structural Changes in People at Risk of or with Mild Knee Osteoarthritis. Prospective Data from the Multicenter Osteoarthritis Study	no high intensity training
32	H. Osteras and F. Paulsberg	2021	The acute hypoalgesic effect of bicycling on pain for patients with knee osteoarthritis: A cohort study	no high intensity training
33	T. Ozmen, U. Gafuroglu, A. Altun Guvenir, I. Ziraman and B. Ozkurt	2017	RELATIONSHIP BETWEEN KINESIOPHOBIA, QUADRICEPS MUSCLE STRENGTH AND QUALITY OF LIFE IN PATIENTS WITH KNEE OSTEOARTHRITIS	no high intensity training
34	B. D. Perez-Huerta, B. Diaz-Pulido, D. Pecos-Martin, D. Beckwee, E. Lluch-Girbes, R. Fernandez-Matias, M. J. B. Rubio and T. Gallego-Izquierdo	2020	Effectiveness of a Program Combining Strengthening, Stretching, and Aerobic Training Exercises in a Standing versus a Sitting Position in Overweight Subjects with Knee Osteoarthritis: A Randomized Controlled Trial	no high intensity training
35	B. A. Rakel, M. B. Zimmerman, K. Geasland, J. Embree, C. R. Clark, N. O. Noiseux, J. J. Callaghan, K. Herr, D. Walsh and K. A. Sluka	2014	Transcutaneous electrical nerve stimulation for the control of pain during rehabilitation after total knee arthroplasty: A randomized, blinded, placebo-controlled trial	no high intensity training
36	J. P. Regnaux, M. M. Lefevre-Colau, L. Trinquart, C. Nguyen, I. Boutron, L. Brosseau and P. Ravaud	2015	High-intensity versus low-intensity physical activity or exercise in people with hip or knee osteoarthritis	no high intensity training
37	S. Rewald, A. F. T. Lenssen, P. J. Emans, R. A. de Bie, G. van Breukelen and I. Mesters	2020	Aquatic Cycling Improves Knee Pain and Physical Functioning in Patients With Knee Osteoarthritis: A Randomized Controlled Trial	no high intensity training
38	M. Rubio-Morales, F. A. Miralles-Muñoz, S. Gonzalez-Parreño, M. Ruiz-Lozano, A. Lizaur-Utrilla and M. F. Vizcaya-Moreno	2023	A relevant number of patients do not increase their engagement in physical, social and leisure activities at the medium-term after total knee arthroplasty: a prospective cohort study	no high intensity training
39	S. Samaan, M. G. Sedhom and M. O. Grace	2022	A randomized comparative study between high-intensity laser vs low-intensity pulsed ultrasound both combined with exercises for the treatment of knee osteoarthritis	no high intensity training
40	P. Siriratna, C. Ratanasutiranont, T. Manissorn, N. Santiniyom and W. Chira-Adisai	2022	Short-Term Efficacy of High-Intensity Laser Therapy in Alleviating Pain in Patients with Knee Osteoarthritis: A Single-Blind Randomised Controlled Trial	no high intensity training

41	B. Skoffer, T. Maribo, I. Mechlenburg, C. G. Korsgaard, K. Søballe and U. Dalgas	2020	Efficacy of preoperative progressive resistance training in patients undergoing total knee arthroplasty: 12-month follow-up data from a randomized controlled trial	no high intensity training
42	S. T. Skou, A. Bricca and E. M. Roos	2018	The impact of physical activity level on the short- and long-term pain relief from supervised exercise therapy and education: a study of 12,796 Danish patients with knee osteoarthritis	no high intensity training
43	M. B. Stausholm, I. F. Naterstad, P. P. Alfredo, C. Couppe, K. V. Fersum, E. C. P. Leal, R. A. B. Lopes-Martins, J. Joensen and J. M. Bjordal	2022	Short- and Long-Term Effectiveness of Low-Level Laser Therapy Combined with Strength Training in Knee Osteoarthritis: A Randomized Placebo-Controlled Trial	no high intensity training
44	N. Stiglić-Rogoznica, D. Stamenković, L. Frlan-Vrgoc, V. Avancini-Dobrović and T. S. Vrbanić	2011	Analgesic effect of high intensity laser therapy in knee osteoarthritis	no high intensity training
45	M. Terradas-Monllor, M. Ochandorena-Acha, H. Beltran-Alacreu, E. G. Oltra, F. C. Saenz and J. H. Hermoso		A feasibility study of home-based preoperative multimodal physiotherapy for patients scheduled for a total knee arthroplasty who catastrophize about their pain	no high intensity training
46	J. Thomazeau, A. Rouquette, V. Martinez, C. Rabuel, N. Prince, J. L. Laplanche, R. Nizard, J. F. Bergmann, S. Perrot and C. Lloret-Linares	2016	Predictive Factors of Chronic Post-Surgical Pain at 6 Months Following Knee Replacement: Influence of Postoperative Pain Trajectory and Genetics	no high intensity training
47	C. S. Thudium, A. Engstrøm, A. C. Bay-Jensen, P. Frederiksen, N. Jansen, A. De Zwart, M. van der Leeden, J. Dekker, W. Lems, L. Roorda, W. E. van Spil and M. Van der Esch	2023	Cartilage tissue turnover increases with high- compared to low-intensity resistance training in patients with knee OA	no high intensity training
48	K. Vader, A. B. Abebe, M. B. Chala, K. Varette and J. Miller	2020	Determining the feasibility of a trial to evaluate the effectiveness of phototherapy versus placebo at reducing pain during physical activity for people with knee osteoarthritis: a pilot randomized controlled trial	no high intensity training
49	D. K. White, C. Tudor-Locke, D. T. Felson, K. D. Gross, J. B. Niu, M. Nevitt, C. E. Lewis, J. Torner and T. Neogi	2013	Do radiographic disease and pain account for why people with or at high risk of knee osteoarthritis do not meet physical activity guidelines?	no high intensity training
50	B. Woods, A. Manca, H. Weatherly, P. Saramago, E. Sideris, C. Giannopoulou, S. Rice, M. Corbett, A. Vickers, M. Bowes, H. MacPherson and M. Sculpher	2017	Cost-effectiveness of adjunct non-pharmacological interventions for osteoarthritis of the knee	no high intensity training
51	S. Y. Ababneh, J. W. Prescott and M. N. Gurcan	2011	Automatic graph-cut based segmentation of bones from knee magnetic resonance images for osteoarthritis research	no KOA
52	A. Agarwalla, D. R. Christian, J. N. Liu, G. H. Garcia, M. L. Redondo, A. B. Yanke and B. J. Cole	2021	Return to Work Following Isolated Opening Wedge High Tibial Osteotomy	no KOA
53	M. S. Akaltun, O. Altindag, N. Turan, S. Gursoy and A. Gur	2021	Efficacy of high intensity laser therapy in knee osteoarthritis: a double-blind controlled randomized study	no KOA
54	A. Armstrong, K. J. Rodriguez, A. Sabag, Y. Mavros, H. M. Parker, S. E. Keating and N. A. Johnson	2022	Effect of aerobic exercise on waist circumference in adults with overweight or obesity: A systematic review and meta-analysis	no KOA
55	N. L. Ashworth, K. E. Chad, E. L. Harrison, B. A. Reeder and S. C. Marshall	2005	Home versus center based physical activity programs in older adults	no KOA
56	J. J. Bjerre-Bastos, C. Sejersen, A. R. Bihlet, N. H. Secher, A. L. Mackey, C. C. Kitchen, P. Drobinski, C. S. Thudium and H. B. Nielsen	2022	An Estimate of Plasma Volume Changes Following Moderate-High Intensity Running and Cycling Exercise and Adrenaline Infusion	no KOA
57	M. R. Bougar, H. A. Veiskarami, S. Khodarahimi, A. Izadpanah, M. Sadeghi and N. Nazari	2022	Effectiveness of Three Physical Treatments on Pain Perception and Emotional State in Males with Chronic Joint Pain	no KOA
58	M. Bridges, J. Hilliard and K. V. Chui	2020	Effects of Light Therapy on Osteoarthritis and Its Sequelae in Aging and Older Adults A Systematic Narrative Review	no KOA
59	N. M. Cattano, J. B. Driban, M. F. Barbe, R. T. Tierney, M. Amin and M. R. Sitler	2017	Biochemical Response to a Moderate Running Bout in Participants With or Without a History of Acute Knee Injury	no KOA
60	Q. Chang and C. L. Huang	2006	Effects of moving training on histology and biomarkers levels of articular cartilage	no KOA

61	Q. Chang, C. L. Huang and Z. F. Huang	2007	Matrix metalloproteinases and inhibitor in knee synovial fluid as cartilage biomarkers in rabbits: The effect of high-intensity jumping exercise	no KOA
62	L. Chen, Y. Ji, X. M. Hu, C. Cui, H. Liu, Y. F. Tang, B. W. Qi, Y. H. Niu, X. Hu, A. X. Yu and Q. L. Fan	2018	Cationic poly-L-lysine-encapsulated melanin nanoparticles as efficient photoacoustic agents targeting to glycosaminoglycans for the early diagnosis of articular cartilage degeneration in osteoarthritis	no KOA
63	Q. Chen, H. Luo, C. C. Zhou, H. Yu, S. Yao, F. D. Fu, R. Seeley, X. Ji, Y. P. Yang, P. F. Chen, H. T. Jin, P. J. Tong, D. Chen, C. L. Wu, W. B. Du and H. F. Ruan	2020	Comparative intra-articular gene transfer of seven adeno-associated virus serotypes reveals that AAV2 mediates the most efficient transduction to mouse arthritic chondrocytes	no KOA
64	S. J. Chen, Y. X. Zhu, W. Y. Lao and Y. L. Zhou	2018	Establishment of a rat model of chronic osteoarthritis by intra-articular injection combined with treadmill exercise	no KOA
65	L. Christensen, N. K. Vollestad, M. B. Veierod, B. Stuge, J. Cabri and H. S. Robinson	2019	The Timed Up & Go test in pregnant women with pelvic girdle pain compared to asymptomatic pregnant and non-pregnant women	no KOA
66	P. G. Conaghan	2002	Update on osteoarthritis part 1: current concepts and the relation to excercise	no KOA
67	C. A. Courtney, R. M. Rine, D. T. Jenk, P. D. Collier and A. Waters	2013	Enhanced Proprioceptive Acuity at the Knee in the Competitive Athlete	no KOA
68	T. J. Crijns, N. Brinkman, S. Ramtin, D. Ring, J. Doornberg, P. Jutte and K. Koenig	2022	Are There Distinct Statistical Groupings of Mental Health Factors and Pathophysiology Severity Among People with Hip and Knee Osteoarthritis Presenting for Specialty Care?	no KOA
69	G. L. Cvetanovich, D. R. Christian, G. H. Garcia, J. N. Liu, M. L. Redondo, A. B. Yanke and B. J. Cole	2020	Return to Sport and Patient Satisfaction After Meniscal Allograft Transplantation	no KOA
70	J. E. Depiazzi, R. A. Forbes, N. Gibson, N. L. Smith, A. C. Wilson, R. N. Boyd and K. Hill	2019	The effect of aquatic high-intensity interval training on aerobic performance, strength and body composition in a non-athletic population: systematic review and meta-analysis	no KOA
71	G. D. Deyle, N. E. Henderson, R. L. Matekel, M. G. Ryder, M. B. Garber and S. C. Allison	2000	Effectiveness of manual physical therapy and exercise in osteoarthritis of the knee. A randomized, controlled trial	no KOA
72	V. T. do Amaral, B. Fernandes, A. Y. Ngomane, I. R. Marcal, G. D. Zanini and E. G. Ciolac	2021	Short-term community-based exercise programs in low-income older women: Does exercise intensity and modality matters?	no KOA
73	L. P. dos Santos, R. C. D. Santo, T. R. Ramis, J. K. S. Portes, R. M. D. Chakr and R. M. Xavier	2021	The effects of resistance training with blood flow restriction on muscle strength, muscle hypertrophy and functionality in patients with osteoarthritis and rheumatoid arthritis: A systematic review with meta-analysis	no KOA
74	B. Fang, Y. H. Kim and M. Y. Choi	2022	Effects of High-Intensity Aquatic or Bicycling Training in Athletes with Unilateral Patellofemoral Pain Syndrome	no KOA
75	A. Finney, K. S. Dziedzic, M. Lewis and E. Healey	2017	Multisite peripheral joint pain: a cross-sectional study of prevalence and impact on general health, quality of life, pain intensity and consultation behaviour	no KOA
76	E. Folkesson, A. Turkiewicz, N. Ali, M. Ryden, H. V. Hughes, J. Tjornstrand, P. Onnerfjord and M. Englund	2020	Proteomic comparison of osteoarthritic and reference human menisci using data-independent acquisition mass spectrometry	no KOA
77	C. E. S. Franciozi, V. A. F. Tarini, R. D. Reginato, P. R. S. Goncalves, V. P. Medeiros, M. Ferretti, J. L. Dreyfuss, H. B. Nader and F. Faloppa	2013	Gradual strenuous running regimen predisposes to osteoarthritis due to cartilage cell death and altered levels of glycosaminoglycans	no KOA
78	Y. Fukumoto, H. Tateuchi, T. Ikezoe, R. Tsukagoshi, H. Akiyama, K. So, Y. Kuroda and N. Ichihashi	2014	Effects of high-velocity resistance training on muscle function, muscle properties, and physical performance in individuals with hip osteoarthritis: A randomized controlled trial	no KOA
79	S. A. Garcia, T. J. Moffit, M. N. Vakula, S. C. Holmes, M. M. Montgomery and D. N. Pamukoff	2020	Quadriceps Muscle Size, Quality, and Strength and Self-Reported Function in Individuals With Anterior Cruciate Ligament Reconstruction	no KOA
80	W. Geidl, K. Abu-Omar, M. Weege, S. Messing and K. Pfeifer	2020	German recommendations for physical activity and physical activity promotion in adults with noncommunicable diseases	no KOA
81	S. Z. George, J. M. Beneciuk, T. A. Lentz, S. S. Wu, Y. Dai, J. E. Bialosky and G. Zeppieri	2018	Optimal Screening for Prediction of Referral and Outcome (OSPRO) for Musculoskeletal Pain Conditions: Results From the Validation Cohort	no KOA

82	D. J. Green, M. Lewis, G. Mansell, M. Artus, K. S. Dziedzic, E. M. Hay, N. E. Foster and D. A. van der Windt	2018	Clinical course and prognostic factors across different musculoskeletal pain sites: A secondary analysis of individual patient data from randomised clinical trials	no KOA
83	V. Gremeaux, J. Renault, L. Pardon, G. Deley, R. Lepers and J. M. Casillas	2008	Low-Frequency Electric Muscle Stimulation Combined With Physical Therapy After Total Hip Arthroplasty for Hip Osteoarthritis in Elderly Patients: A Randomized Controlled Trial	no KOA
84	Y. Guerrero, N. Soomro, G. Wilson, Y. Dam, J. Meiklejohn, K. Simpson, R. Smith, J. Brand-Miller, M. Simic, H. O'Connor, Y. Mavros, N. Foroughi, T. Poon, K. Bradshaw, L. March, B. Vanwanseele, F. Eckstein, M. Fransen, J. Bergamasco, A. Anandacoomarasamy and M. F. Singh	2015	Train High Eat Low for Osteoarthritis study (THE LO study): protocol for a randomized controlled trial	no KOA
85	M. Guillot, M. Moreau, M. Heit, J. Martel-Pelletier, J. P. Pelletier and E. Troncy	2013	m Characterization of osteoarthritis in cats and meloxicam efficacy using objective chronic pain evaluation tools	no KOA
86	L. E. Hart, D. A. Haaland, D. A. Baribeau, I. M. Mukovozov and T. F. Sabljic	2008	The Relationship Between Exercise and Osteoarthritis in the Elderly	no KOA
87	S. Hasan, A. Alonazi, S. Anwer, A. Jamal, S. Parvez, F. A. S. Alfaiz and H. Li	2022	Efficacy of Patellar Taping and Electromyographic Biofeedback Training at Various Knee Angles on Quadriceps Strength and Functional Performance in Young Adult Male Athletes with Patellofemoral Pain Syndrome: A Randomized Controlled Trial	no KOA
88	E. B. Hassan, M. Mirams, A. Ghasem-Zadeh, E. J. Mackie and R. C. Whitton	2016	Role of subchondral bone remodelling in collapse of the articular surface of Thoroughbred racehorses with palmar osteochondral disease	no KOA
89	M. A. Holden, P. J. A. Nicolson, M. J. Thomas, N. Corp, R. S. Hinman and K. L. Bennell	2023	Osteoarthritis year in review 2022: rehabilitation	no KOA
90	J. M. Hootman, C. A. Macera, C. G. Helmick and S. N. Blair	2003	Influence of physical activity-related joint stress on the risk of self-reported hip/knee osteoarthritis: a new method to quantify physical activity	no KOA
91	L. Hughes, B. Paton, F. Haddad, B. Rosenblatt, C. Gissane and S. D. Patterson	2018	Comparison of the acute perceptual and blood pressure response to heavy load and light load blood flow restriction resistance exercise in anterior cruciate ligament reconstruction patients and non-injured populations	no KOA
92	A. H. Jaffri, M. Lynch, S. A. Saliba and J. M. Hart	2021	Quadriceps Oxygenation During Exercise in Patients With Anterior Cruciate Ligament Reconstruction	no KOA
93	J. L. Jaremko, R. W. Cheng, R. G. Lambert, A. F. Habib and J. L. Ronsky	2006	Reliability of an efficient MRI-based method for estimation of knee cartilage volume using surface registration	no KOA
94	D. L. Jones	2011	A public health perspective on physical activity after total hip or knee arthroplasty for osteoarthritis	no KOA
95	A. B. Jønsson, C. V. Johansen, N. Rolving and M. Pfeiffer-Jensen	2021	Feasibility and estimated efficacy of blood flow restricted training in female patients with rheumatoid arthritis: a randomized controlled pilot study	no KOA
96	C. O. Kean, T. B. Birmingham, S. J. Garland, D. M. Bryant and J. R. Giffin	2011	Preoperative strength training for patients undergoing high tibial osteotomy: a prospective cohort study with historical controls	no KOA
97	Y. A. Kharaz, H. Birch, A. Chester, E. Alchorne, D. Simpson, P. Clegg and E. Comerford	2021	The effect of exercise on the protein profile of rat knee joint intra- and extra-articular ligaments	no KOA
98	K. M. Knutzen, B. A. Pendergrast, B. Lindsey and L. R. Brilla	2007	The effect of high resistance weight training on reported pain in older adults	no KOA
99	T. A. Lentz, S. Z. George, O. Manickas-Hill, M. R. Malay, J. O'Donnell, P. Jayakumar, W. Jiranek and R. C. Mather	2020	What General and Pain-associated Psychological Distress Phenotypes Exist Among Patients with Hip and Knee Osteoarthritis?	no KOA
100	S. Q. Li, S. Shaharudin and M. R. A. Kadir	2021	Effects of Blood Flow Restriction Training on Muscle Strength and Pain in Patients With Knee Injuries A Meta-Analysis	no KOA
101	X. Li, W. Pan, H. Qin, L. Qu, H. Zhang and X. Zhu	2019	Blood flow restriction training: A new method for accelerating musculoskeletal rehabilitation	no KOA

102	C. D. Liao, Y. T. Wu, J. Y. Tsauo, P. R. Chen, Y. K. Tu, H. C. Chen and T. H. Liou	2020	Effects of protein supplementation combined with exercise training on muscle mass and function in older adults with lower-extremity osteoarthritis: A systematic review and meta-analysis of randomized trials	no KOA
103	A. R. Lieverse, B. Mack, V. I. Bazaliiskii and A. W. Weber	2016	Revisiting osteoarthritis in the Cis-Baikal: Understanding behavioral variability and adaptation among middle Holocene foragers	no KOA
104	L. Liu, H. Liu, Z. Zhen, Y. Zheng, X. Zhou, E. Raithel, J. Du, Y. Hu, W. Chen and X. Hu	2022	Analysis of Knee Joint Injury Caused by Physical Training of Freshmen Students Based on 3T MRI and Automatic Cartilage Segmentation Technology: A Prospective Study	no KOA
105	L. P. Liu, N. Chang, S. H. Li, P. P. Gong and J. H. Wang	2022	Optical Microscope Rehabilitation Nursing Study of Anterior Cruciate Ligament Injury through Lateral Knee Incision Based on Medical Internet of Things	no KOA
106	H. S. Longpre, J. R. Potvin and M. R. Maly	2013	Biomechanical changes at the knee after lower limb fatigue in healthy young women	no KOA
107	J. F. Lopez, R. V. Espinoza, F. Alvear-Vasquez, L. S. Macedo, D. M. Velasquez, W. R. Pacco, M. C. Bolaños and R. G. Campos	2021	Systematic review of aquatic physical exercise programs on functional fitness in older adults	no KOA
108	S. Lucarno, M. Zago, M. Buckthorpe, A. Grassi, F. Tosarelli, R. Smith and F. Della Villa	2021	Systematic Video Analysis of Anterior Cruciate Ligament Injuries in Professional Female Soccer Players	no KOA
109	V. A. Lukas, K. W. Fishbein, P. C. Lin, M. Schar, E. Schneider, C. P. Neu, R. G. Spencer and D. A. Reiter	2015	Classification of Histologically Scored Human Knee Osteochondral Plugs by Quantitative Analysis of Magnetic Resonance Images at 3T	no KOA
110	N. Magni, P. McNair and D. Rice	2022	Six weeks of resistance training (plus advice) vs advice only in hand osteoarthritis: A single-blind, randomised, controlled feasibility trial	no KOA
111	C. D. Mallen, B. I. Nicholl, M. Lewis, B. Bartlam, D. Green, S. Jowett, J. Kigozi, J. Belcher, K. Clarkson, Z. Lingard, C. Pope, C. A. Chew-Graham, P. Croft, E. M. Hay and G. Peat	2017	The effects of implementing a point-of-care electronic template to prompt routine anxiety and depression screening in patients consulting for osteoarthritis (the Primary Care Osteoarthritis Trial): A cluster randomised trial in primary care	no KOA
112	T. Manyanga, M. Froese, R. Zarychanski, A. Abou-Setta, C. Friesen, M. Tennenhouse and B. L. Shay	2014	Pain management with acupuncture in osteoarthritis: a systematic review and meta-analysis	no KOA
113	K. A. Martin and A. R. Sinden	2001	Who will stay and who will go? A review of older adults' adherence to randomized controlled trials of exercise	no KOA
114	J. S. Mason, M. S. Crowell, R. A. Brindle, J. A. Dolbeer, E. M. Miller, T. A. Telemeco and D. L. Goss	2022	The Effect of Blood Flow Restriction Training on Muscle Atrophy Following Meniscal Repair or Chondral Restoration Surgery in Active Duty Military: A Randomized Controlled Trial	no KOA
115	J. L. Mateer, J. M. Hoch, C. G. Mattacola, T. A. Butterfield and C. Lattermann	2015	Serum Cartilage Oligomeric Matrix Protein Levels in Collegiate Soccer Athletes over the Duration of an Athletic Season: A Pilot Study	no KOA
116	F. Mayer, H. Schmitt and H. H. Dickhuth	2003	The relevance of sports for the onset, prevention and rehabilitation of osteoarthritis	no KOA
117	J. A. Mettler, D. M. Magee and B. M. Doucet	2018	High-Frequency Neuromuscular Electrical Stimulation Increases Anabolic Signaling	no KOA
118	G. Milandri and S. Sivarasu	2021	A Randomized Controlled Trial of Eccentric Versus Concentric Cycling for Anterior Cruciate Ligament Reconstruction Rehabilitation	no KOA
119	J. J. Mitchell, J. Chahla and R. F. LaPrade	2016	Editorial Commentary: Limited Data Shows How Little We Know	no KOA
120	H. Moffet, L. Noreau, E. Parent and M. Drolet	2000	Feasibility of an eight-week dance-based exercise program and its effects on locomotor ability of persons with functional class III rheumatoid arthritis	no KOA
121	M. Moreau, B. Lussier, J. P. Pelletier, J. Martel-Pelletier, C. Bedard, D. Gauvin and E. Troncy	2012	Brachystemma calycinum D. Don Effectively Reduces the Locomotor Disability in Dogs with Naturally Occurring Osteoarthritis: A Randomized Placebo-Controlled Trial	no KOA
122	P. J. Mork, A. Holtermann and T. I. Nilsen	2012	Effect of body mass index and physical exercise on risk of knee and hip osteoarthritis: longitudinal data from the Norwegian HUNT Study	no KOA
123	S. L. Murphy, D. M. Strasburg, A. K. Lyden, D. M. Smith, J. F. Koliba, D. P. Dadabhoy and S. M. Wallis	2008	Effects of Activity Strategy Training on Pain and Physical Activity in Older Adults With Knee or Hip Osteoarthritis: A Pilot Study	no KOA

124	R. C. Murray, H. L. Birch, K. Lakhani and A. E. Goodship	2001	Biochemical composition of equine carpal articular cartilage is influenced by short-term exercise in a site-specific manner	no KOA
125	R. C. Murray, C. F. Zhu, A. E. Goodship, K. H. Lakhani, C. M. Agrawal and K. A. Athanasiou	1999	Exercise affects the mechanical properties and histological appearance of equine articular cartilage	no KOA
126	W. N. Newberry, J. J. Garcia, C. D. Mackenzie, C. E. Decamp and R. C. Haut	1998	Analysis of acute mechanical insult in an animal model of post-traumatic osteoarthrosis	no KOA
127	G. X. Ni, L. Q. Zhan, M. Q. Gao, L. Lei, Y. Z. Zhou and Y. X. Pan	2011	Matrix metalloproteinase-3 inhibitor retards treadmill running-induced cartilage degradation in rats	no KOA
128	B. J. Nicklas, W. Ambrosius, S. P. Messier, G. D. Miller, B. Penninx, R. F. Loeser, S. Palla, E. Bleecker and M. Pahor	2004	Diet-induced weight loss, exercise, and chronic inflammation in older, obese adults: a randomized controlled clinical trial	no KOA
129	J. Nyland, A. Huffstutler, J. Faridi, S. Sachdeva, M. Nyland and D. Caborn	2020	Cruciate ligament healing and injury prevention in the age of regenerative medicine and technostress: homeostasis revisited	no KOA
130	M. C. O'Dell, D. Jaramillo, L. Bancroft, L. Varich, G. Logsdon and S. Servaes	2016	Imaging of Sports-related Injuries of the Lower Extremity in Pediatric Patients	no KOA
131	H. S. Park, J. S. Song and E. K. Kim	2022	Effects of low-intensity resistance exercise with blood flow restriction after high tibial osteotomy in middle-aged women	no KOA
132	J. C. Patzkowski, J. G. Owens, R. V. Blanck, K. L. Kirk and J. R. Hsu	2012	Deployment after limb salvage for high-energy lower-extremity trauma	no KOA
133	C. Qi and H. Changlin	2007	Levels of biomarkers correlate with magnetic resonance imaging progression of knee cartilage degeneration: a study on canine	no KOA
134	C. Qi and H. Changlin	2008	EFFECTS OF DIFFERENT MODE RIGH-INTENSITY MOVEMENT TRAINING ON ARTICULAR CARTILAGE IN HISTOLOGY - A RANDOMIZED CONTROLLED TRIAL ON RABBIT KNEE	no KOA
135	S. Raman, G. E. Gold, M. S. Rosen and B. Sveinsson	2022	Automatic estimation of knee effusion from limited MRI data	no KOA
136	W. J. Rejeski, R. Axtell, R. Fielding, J. Katula, A. C. King, T. M. Manini, A. P. Marsh, M. Pahor, A. Rego, C. Tudor-Locke, M. Newman, M. P. Walkup, M. E. Miller and L. S. I. Grp	2013	Promoting physical activity for elders with compromised function: the Lifestyle Interventions and Independence for Elders (LIFE) Study physical activity intervention	no KOA
137	J. L. Rios, K. R. Boldt, J. W. Mather, R. A. Seerattan, D. A. Hart and W. Herzog	2018	Quantifying the Effects of Different Treadmill Training Speeds and Durations on the Health of Rat Knee Joints	no KOA
138	R. Rodrigues, R. B. Ferraz, C. O. Kurimori, L. K. Guedes, F. R. Lima, A. L. de Sa-Pinto, B. Gualano and H. Roschel	2020	Low-Load Resistance Training With Blood-Flow Restriction in Relation to Muscle Function, Mass, and Functionality in Women With Rheumatoid Arthritis	no KOA
139	F. W. Roemer, A. Guermazi, Y. Q. Zhang, M. Yang, D. J. Hunter, M. D. Crema and K. Bohndorf	2009	Hoffa's Fat Pad: Evaluation on Unenhanced MR Images as a Measure of Patellofemoral Synovitis in Osteoarthritis	no KOA
140	S. Rolff, C. Korallus and A. A. Hanke	2020	Rehabilitation with the aid of blood flow restriction training	no KOA
141	A. Sarmanova, M. Doherty, C. Kuo, J. Wei, A. Abhishek, C. Mallen, C. Zeng, Y. Wang, G. Lei and W. Zhang	2020	Statin use and risk of joint replacement due to osteoarthritis and rheumatoid arthritis: a propensity-score matched longitudinal cohort study	no KOA
142	R. Schachar and D. Ogilvie-Harris	2015	Osteoarthritis: Joint conservation strategies	no KOA
143	J. M. Schulz, T. B. Birmingham, H. F. Atkinson, E. Woehrle, C. A. Primeau, M. J. Lukacs, B. K. Al-Khazraji, M. C. M. Khan, B. O. Zomar, R. J. Petrella, F. Beier, C. T. Appleton, J. K. Shoemaker and D. M. Bryant	2020	Are we missing the target? Are we aiming too low? What are the aerobic exercise prescriptions and their effects on markers of cardiovascular health and systemic inflammation in patients with knee osteoarthritis? A systematic review and meta-analysis	no KOA

144	A. I. Semciw, T. Pizzari, S. Woodley, A. Zacharias, M. Kingsley and R. A. Green	2018	Targeted gluteal exercise versus sham exercise on self-reported physical function for people with hip osteoarthritis (the GHOst trial - Gluteal exercise for Hip Osteoarthritis): a protocol for a randomised clinical trial	no KOA
145	M. Sengupta, Y. Q. Zhang, J. B. Niu, A. Guermazi, M. Grigorian, D. Gale, D. T. Felson and D. J. Hunter	2006	High signal in knee osteophytes is not associated with knee pain	no KOA
146	J. D. Sessford, L. R. Brawley and N. C. Gyurcsik	2015	Examination of Self-Regulatory Efficacy and Pain Among Individuals Challenged by Arthritis Flares	no KOA
147	D. Shin	2014	Association Between Metabolic Syndrome, Radiographic Knee Osteoarthritis, and Intensity of Knee Pain: Results of a National Survey	no KOA
148	A. C. Skelly, R. Chou, J. R. Dettori, J. A. Turner, J. L. Friedly, S. D. Rundell, R. Fu, E. D. Brodt, N. Wasson, C. Winter and A. J. R. Ferguson	2018	AHRQ Comparative Effectiveness Reviews	no KOA
149	M. Sobejana, J. van den Hoek, G. S. Metsios, G. D. Kitas, M. van der Leeden, S. Verberne, H. T. Jorstad, M. Pijnappels, W. F. Lems, M. T. Nurmohamed and M. van der Esch	2022	Exercise intervention on cardiorespiratory fitness in rheumatoid arthritis patients with high cardiovascular disease risk: a single-arm pilot study	no KOA
150	J. D. Sprick and C. A. Rickards	2017	Cyclical blood flow restriction resistance exercise: a potential parallel to remote ischemic preconditioning?	no KOA
151	B. Tanriverdi, G. Tanriverdi, E. Ercin, F. K. Dagistanli, H. N. Ones, E. Edipoglu, S. D. Delipinar, B. Isildar and M. G. Bilgili	2021	THE FAT PAT TISSUE EXPRESSES A PATHOGENIC PROFILE OF INFLAMMATORY MEDIATORIES AND 4-HNE IN PATIENTS WITH OSTEOARTHRITIS	no KOA
152	J. L. W. Taylor, C. M. Campbell, R. J. Thorpe, K. E. Whitfield, M. Nkimbeng and S. L. Szanton	2018	Pain, Racial Discrimination, and Depressive Symptoms among African American Women	no KOA
153	C. C. Tomás, E. Oliveira, D. Sousa et al.	2016	Proceedings of the 3rd IPLeiria's International Health Congress : Leiria, Portugal. 6-7 May 2016	no KOA
154	K. Townsend, I. Imbert, V. Eaton, G. W. Stevenson and T. King	2022	Voluntary exercise blocks ongoing pain and diminishes bone remodeling while sparing protective mechanical pain in a rat model of advanced osteoarthritis pain	no KOA
155	M. Van der Esch, M. Heijmans and J. Dekker	2003	Factors contributing to possession and use of walking aids among persons with rheumatoid arthritis and osteoarthritis	no KOA
156	H. K. Vincent and K. R. Vincent	2013	Considerations for Initiating and Progressing Running Programs in Obese Individuals	no KOA
157	V. Wylde, V. Wells, S. Dixon and R. Gooberman-Hill	2014	The colour of pain: can patients use colour to describe osteoarthritis pain?	no KOA
158	T. Yasuda	2022	Selected Methods of Resistance Training for Prevention and Treatment of Sarcopenia	no KOA
159	X. C. Yuan, B. Zhu, X. H. Jing, L. Z. Xiong, C. H. Wu, F. Gao, H. P. Li, H. C. Xiang, H. Zhu, B. Zhou, W. He, C. Y. Lin, H. L. Pan, Q. Wang and M. Li	2018	Electroacupuncture Potentiates Cannabinoid Receptor-Mediated Descending Inhibitory Control in a Mouse Model of Knee Osteoarthritis	no KOA
160	L. A. Zdziarski, J. G. Wasser and H. K. Vincent	2015	Chronic pain management in the obese patient: a focused review of key challenges and potential exercise solutions	no KOA
161	N. Zeng, T. Liao, X. Y. Chen, Z. P. Yan, J. T. Li and G. X. Ni	2021	Treadmill running induces remodeling of the infrapatellar fat pad in an intensity-dependent manner	no KOA
162	R. Zhang	2022	Application of Biomaterials in Tendon Injury Healing and Adhesion in Sports	no KOA
163	X. Zhou, H. Cao, M. Wang, J. Zou and W. Wu	2021	Moderate-intensity treadmill running relieves motion-induced post-traumatic osteoarthritis mice by up-regulating the expression of lncRNA H19	no KOA
164	O. Abrahin, R. P. Rodrigues, E. C. Sousa, J. D. Beas-Jiménez, A. C. Marçal and M. E. Da Silva-Grigoletto	2015	Effect of 24 sessions of resistance training in a patient with gonarthrosis bilateral: A case study	no cohort/RCT
165	A. Agarwalla, J. N. Liu, H. H. Wu, I. L. Kalbian, G. H. Garcia and B. E. Shubin Stein	2021	Return to Work Following Tibial Tubercle Osteotomy for Patellofemoral Osteoarthritis and Pain	no cohort/RCT

166	S. R. Akinbo, O. Sokunbi and T. Ogunbameru	2008	Factors contributing to possession of walking aids among persons with osteoarthritis of the knee	no cohort/RCT
167	M. S. M. Alayat, T. H. A. Aly, A. E. M. Elsayed and A. S. M. Fadil	2017	Efficacy of pulsed Nd:YAG laser in the treatment of patients with knee osteoarthritis: a randomized controlled trial	no cohort/RCT
168	E. Alentorn-Geli, K. Samuelsson, V. Musahl, C. L. Green, M. Bhandari and J. Karlsson	2017	The Association of Recreational and Competitive Running With Hip and Knee Osteoarthritis: A Systematic Review and Meta-analysis	no cohort/RCT
169	M. J. Bade and J. E. Stevens-Lapsley	2012	Restoration of physical function in patients following total knee arthroplasty: an update on rehabilitation practices	no cohort/RCT
170	S. M. Bierma-Zeinstra and B. W. Koes	2007	Risk factors and prognostic factors of hip and knee osteoarthritis	no cohort/RCT
171	H. Bonakdari, G. Tardif, F. Abram, J. P. Pelletier and J. Martel-Pelletier	2020	Serum adipokines/related inflammatory factors and ratios as predictors of infrapatellar fat pad volume in osteoarthritis: Applying comprehensive machine learning approaches	no cohort/RCT
172	L. Brosseau, L. MacLeay, V. Robinson, G. Wells and P. Tugwell	2003	Intensity of exercise for the treatment of osteoarthritis	no cohort/RCT
173	L. Brosseau, J. Taki, B. Desjardins, O. Thevenot, M. Fransen, G. A. Wells, A. M. Imoto, K. Toupin-April, M. Westby, I. C. A. Gallardo, W. Gifford, L. Laferriere, P. Rahman, L. Loew, G. De Angelis, S. Cavallo, S. M. Shallwani, A. Aburub, K. L. Bennell, M. Van der Esch, M. Simic, S. McConnell, A. Harmer, G. P. Kenny, G. Paterson, J. P. Regnaux, M. M. Lefevre-Colau and L. McLean	2017	The Ottawa panel clinical practice guidelines for the management of knee osteoarthritis. Part three: aerobic exercise programs	no cohort/RCT
174	M. S. Cerqueira and W. H. D. Vieira	2019	Effects of blood flow restriction exercise with very low load and low volume in patients with knee osteoarthritis: protocol for a randomized trial	no cohort/RCT
175	A. C. De Almeida, M. G. Pedroso, J. B. Aily, G. H. Gonçalves, C. M. Pastre and S. M. Mattiello	2018	Influence of a periodized circuit training protocol on intermuscular adipose tissue of patients with knee osteoarthritis: Protocol for a randomized controlled trial	no cohort/RCT
176	H. A. de Ruiter, B. van Gorp and J. F. A. Eijkenboom	2017	Comments on "Effects of high intensity resistance aquatic training on body composition and walking speed in women with mild knee osteoarthritis: a 4-month RCT with 12-month follow-up"	no cohort/RCT
177	L. di Biase, E. Falato, M. L. Caminiti, P. M. Pecoraro, F. Narducci and V. Di Lazzaro	2021	Focused Ultrasound (FUS) for Chronic Pain Management: Approved and Potential Applications	no cohort/RCT
178	W. H. Ettinger and R. F. Afable	1994	PHYSICAL-DISABILITY FROM KNEE OSTEOARTHRITIS - THE ROLE OF EXERCISE AS AN INTERVENTION	no cohort/RCT
179	S. A. M. Fenton, T. Neogi, D. Dunlop, M. Nevitt, M. Doherty, J. L. Duda, R. Klocke, A. Abhishek, A. Rushton, W. Zhang, C. E. Lewis, J. Torner, G. Kitas, D. K. White and G. Multictr Osteoarthritis	2018	Does the intensity of daily walking matter for protecting against the development of a slow gait speed in people with or at high risk of knee osteoarthritis? An observational study	no cohort/RCT
180	M. Fransen, S. McConnell, G. Hernandez-Molina and S. Reichenbach	2014	Exercise for osteoarthritis of the hip	no cohort/RCT
181	W. F. Gomes, A. C. R. Lacerda, G. E. A. Brito-Melo, S. F. Fonseca, E. Rocha-Vieira, A. A. O. Leopoldino, M. R. Amorim and V. A. Mendonca	2016	Aerobic training modulates T cell activation in elderly women with knee osteoarthritis	no cohort/RCT
182	X. H. Guo, P. Zhao, X. Zhou, J. L. Wang and R. R. Wang	2022	A recommended exercise program appropriate for patients with knee osteoarthritis: A systematic review and meta-analysis	no cohort/RCT
183	J. Hua, L. Sun and Y. Teng	2023	Effects of High-Intensity Strength Training in Adults with Knee Osteoarthritis: A Systematic Review and Meta-analysis of Randomized Controlled Trials	no cohort/RCT
184	D. J. Hunter, Y. Q. Zhang, J. B. Niu, J. Goggins, S. Amin, M. P. LaValley, A. Guermazi, H. Genant, D. Gale and D. T. Felson	2006	Increase in bone marrow lesions associated with cartilage loss - A longitudinal magnetic resonance imaging study of knee osteoarthritis	no cohort/RCT

185	P. N. Huu, D. N. Thanh, T. L. T. Hai, H. C. Duc, H. P. Viet and C. N. Trong	2022	Detection and Classification Knee Osteoarthritis Algorithm using YOLOv3 and VGG-16 Models	no cohort/RCT
186	R. A. C. Jardim, T. S. de Sousa, W. N. N. Dos Santos, A. P. Matos and N. C. R. Iosimuta	2022	Blood flow restriction with different load levels in patients with knee osteoarthritis: protocol of a randomized controlled trial	no cohort/RCT
187	J. W. L. Keogh, J. Grigg and C. J. Vertullo	2017	Is Home-Based, High-Intensity Interval Training Cycling Feasible and Safe for Patients With Knee Osteoarthritis?: Study Protocol for a Randomized Pilot Study	no cohort/RCT
188	M. M. Y. Kwok, S. S. M. Ng, S. S. Man and B. C. L. So	2022	The effect of aquatic High Intensity Interval Training on cardiometabolic and physical health markers in women: A systematic review and meta-analysis	no cohort/RCT
189	B. N. Leitzelar and K. F. Koltyn	2021	Exercise and Neuropathic Pain: A General Overview of Preclinical and Clinical Research	no cohort/RCT
190	M. Lewek, J. Stevens and L. Snyder-Mackler	2001	The use of electrical stimulation to increase quadriceps femoris muscle force in an elderly patient following a total knee arthroplasty	no cohort/RCT
191	R. Li, B. Hu, Z. Liu, S. Xu, J. Li, S. Ma, Z. Wang and J. Liu	2021	Insight Into the Effect of Hospital-Based Prehabilitation on Postoperative Outcomes in Patients With Total Knee Arthroplasty: A Retrospective Comparative Study	no cohort/RCT
192	X. D. Li, G. F. Sun, W. B. Zhu and Y. H. Wang	2015	Effects of high intensity exhaustive exercise on SOD, MDA, and NO levels in rats with knee osteoarthritis	no cohort/RCT
193	Y. Liu, Y. Wang, Y. Wang and X. Jia	2022	A Meta-Analysis of Analgesic Effect of Ultrasound Therapy for Patients With Knee Osteoarthritis	no cohort/RCT
194	A. Malorgio, M. Malorgio, M. Benedetti, S. Casarosa and R. Cannataro	2021	High intensity resistance training as intervention method to knee osteoarthritis	no cohort/RCT
195	I. Mechlenburg, B. Skoffer and U. Dalgas	2018	Comparison of Early High-Intensity and Low-Intensity Rehabilitation After Total Knee Arthroplasty: Comment on the Article by Bade et al	no cohort/RCT
196	S. P. Messier	2021	High-Intensity Strength Training, Knee Pain, and Knee Joint Compressive Forces in Adults With Knee Osteoarthritis-Reply	no cohort/RCT
197	S. P. Messier, S. L. Mihalko, D. P. Beavers, B. J. Nicklas, P. DeVita, J. J. Carr, D. J. Hunter, J. D. Williamson, K. L. Bennell, A. Guermazi, M. Lyles and R. F. Loeser	2013	Strength Training for Arthritis Trial (START): design and rationale	no cohort/RCT
198	F. A. Pinheiro, F. O. Pires, B. R. Rønnestad, F. Hardt, M. S. Conceição, M. E. Lixandrão, R. Berton and V. Tricoli	2022	The Effect of Low-intensity Aerobic Training Combined with Blood Flow Restriction on Maximal Strength, Muscle Mass, and Cycling Performance in a Cyclist with Knee Displacement	no cohort/RCT
199	A. Pitsillides, D. Stasinopoulos and I. Mamais	2021	Blood flow restriction training in patients with knee osteoarthritis: Systematic review of randomized controlled trials	no cohort/RCT
200	R. Restuccia, D. Ruggieri, L. Magaudda and R. Talotta	2022	The preventive and therapeutic role of physical activity in knee osteoarthritis	no cohort/RCT
201	S. R. Robbins, L. R. S. Melo, H. Urban, L. A. Deveza, R. Asher, V. L. Johnson and D. J. Hunter	2017	Stepped care approach for medial tibiofemoral osteoarthritis (STrEAMline): Protocol for a randomised controlled trial	no cohort/RCT
202	L. L. Shen, G. F. Huang, W. Tian, L. L. Yu, X. C. Yuan, Z. Q. Zhang, J. Yin, C. Y. Ma, G. W. Cai, J. W. Li, M. Q. Ding, W. He, X. Y. Gao, B. Zhu, X. H. Jing and M. Li	2015	Electroacupuncture inhibits chronification of the acute pain of knee osteoarthritis: study protocol for a randomized controlled trial	no cohort/RCT
203	H. J. Song, H. J. Seo and D. Kim	2020	Effectiveness of high-intensity laser therapy in the management of patients with knee osteoarthritis: A systematic review and meta-analysis of randomized controlled trials	no cohort/RCT
204	D. T. Thomas, R. Shruthi, A. J. Prabhakar, P. V. Dineshbhai and C. Eapen	2022	Hip abductor strengthening in patients diagnosed with knee osteoarthritis – a systematic review and meta-analysis	no cohort/RCT
205	N. Vogel, R. Kaelin, T. Rychen and M. P. Arnold	2022	The German version of the High-Activity Arthroplasty Score is valid and reliable for patients after total knee arthroplasty	no cohort/RCT
206	B. Waller, M. Munukka, U. M. Kujala and A. O. Heinonen	2017	Response to the comments on "Effects of high intensity aquatic resistance training on body composition and walking speed in women with mild knee osteoarthritis: a 4-month RCT with 12-month follow-up"	no cohort/RCT

207	D. K. White, C. Tudor-Locke, D. T. Pelson, K. D. Gross, J. B. Niu, M. Nevitt, C. E. Lewis, J. Torner and T. Neogi	2013	Walking to Meet Physical Activity Guidelines in Knee Osteoarthritis: Is 10,000 Steps Enough?	no cohort/RCT
208	M. Wu, L. Luan, A. Pranata, J. Witchalls, R. Adams, J. Bousie and J. Han	2022	Is high intensity laser therapy more effective than other physical therapy modalities for treating knee osteoarthritis? A systematic review and network meta-analysis	no cohort/RCT
209	J. Wyszyńska and M. Bal-Bocheńska	2018	Efficacy of High-Intensity Laser Therapy in Treating Knee Osteoarthritis: A First Systematic Review	no cohort/RCT
210	A. Zacharias, R. A. Green, A. I. Semciw, M. I. C. Kingsley and T. Pizzari	2014	Efficacy of rehabilitation programs for improving muscle strength in people with hip or knee osteoarthritis: A systematic review withmeta-analysis	no cohort/RCT
211	F. Zhang, J. Kumm, F. Svensson, A. Turkiewicz, R. Frobell and M. Englund	2016	Risk factors for meniscal body extrusion on MRI in subjects free of radiographic knee osteoarthritis: longitudinal data from the Osteoarthritis Initiative	no cohort/RCT
212	J. Zhu, W. Chen, Y. Hu, Y. Qu, H. Yang, Y. Zeng, C. Hou, F. Ge, Z. Zhou and H. Song	2022	Physical activity patterns, genetic susceptibility, and risk of hip/knee osteoarthritis: a prospective cohort study based on the UK Biobank	no cohort/RCT
213	J. Casaña, J. Calatayud, Y. Ezzatvar, J. Vinstrup, J. Benítez and L. L. Andersen	2019	Preoperative high-intensity strength training improves postural control after TKA: randomized-controlled trial	other outcome measures
214	A. H. de Zwart, J. Dekker, L. D. Roorda, M. van der Esch, P. Lips, N. M. van Schoor, A. C. Heijboer, F. Turkstra, M. Gerritsen, A. Hakkinen, K. Bennell, M. Steultjens, W. F. Lems and M. van der Leeden	2020	THE EFFECT OF HIGH-INTENSITY RESISTANCE TRAINING AND VITAMIN D SUPPLEMENTATION ON MUSCLE STRENGTH IN PATIENTS WITH KNEE OSTEOARTHRITIS: A RANDOMIZED CONTROLLED TRIAL	other outcome measures
215	N. E. Jansen, A. Engstrom, C. S. Thudium, A. C. Bay-Jensen, A. H. de Zwart, J. Dekker, W. F. Lems, M. Gerritsen, W. E. van Spil, R. T. Jaspers, F. Turkstra, M. van der Leeden and M. van der Esch	2020	CHANGES IN INFLAMMATION AND MUSCULOSKELETAL TISSUE-DERIVED BIOMARKER SERUM LEVELS IN RESPONSE TO HIGH- AND LOW-INTENSITY RESISTANCE TRAINING IN INDIVIDUALS WITH KNEE OSTEOARTHRITIS	other outcome measures
216	M. J. Bade and J. E. Stevens-Lapsley	2011	Early high-intensity rehabilitation following total knee arthroplasty improves outcomes	unclear exercise principles
217	M. J. Bade, T. Struessel, M. Dayton, J. Foran, R. H. Kim, T. Miner, P. Wolfe, W. M. Kohrt, D. Dennis and J. E. Stevens-Lapsley	2017	Early High-Intensity Versus Low-Intensity Rehabilitation After Total Knee Arthroplasty: A Randomized Controlled Trial	unclear exercise principles
218	K. R. Baker, M. E. Nelson, D. T. Felson, J. E. Layne, R. Sarno and R. Roubenoff	2001	The efficacy of home based progressive strength training in older adults with knee osteoarthritis: a randomized controlled trial	unclear exercise principles
219	Z. de Jong, M. Munneke, A. H. Zwinderman, H. M. Kroon, A. Jansen, K. H. Ronday, D. van Schaardenburg, B. A. C. Dijkmans, C. H. M. Van den Ende, F. C. Breedveld, T. Vlieland and J. M. W. Hazes	2003	Is a long-term high-intensity safe in patients with exercise program effective and rheumatoid arthritis? Results of a randomized controlled trial	unclear exercise principles
220	J. N. Farr, S. B. Going, P. E. McKnight, S. Kasle, E. C. Cussler and M. Cornett	2010	Progressive Resistance Training Improves Overall Physical Activity Levels in Patients With Early Osteoarthritis of the Knee: A Randomized Controlled Trial	unclear exercise principles
221	S. Handa, Y. Kamijo, T. Yamazaki, H. Genno and H. Nose	2009	THE EFFECTS OF HIGH-INTENSITY INTERVAL WALKING TRAINING WITH WATER IMMERSION IN MIDDLE-AGED AND OLDER WOMEN WITH LIGHT KNEE OSTEOARTHRITIS	unclear exercise principles
222	S. Handa, S. Masuki, T. Ohshio, Y. Kamijo, A. Takamata and H. Nose	2016	Target intensity and interval walking training in water to enhance physical fitness in middle-aged and older women: a randomised controlled study	unclear exercise principles
223	K. E. M. Harmelink, R. Dandis, P. J. der Van der Wees Pj, A. Zeegers, M. W. N. der Sanden and J. B. Staal	2021	Recovery trajectories over six weeks in patients selected for a high-intensity physiotherapy program after Total knee Arthroplasty: a latent class analysis	unclear exercise principles
224	T. Hashizaki, Y. Nishimura, T. Ogawa, C. Ohno, K. Kouda, Y. Umemoto, T. Taniguchi, H. Yamada and F. Tajima	2023	Effectiveness of a 3-Week Rehabilitation Program Combining Muscle Strengthening and Endurance Exercises Prior to Total Knee Arthroplasty: A Non-Randomized Controlled Trial	unclear exercise principles
225	P. M. Holm, P. Aagaard, H. Schroder and S. T. Skou	2017	HIGH-INTENSITY STRENGTH TRAINING IN ADDITION TO STANDARD NON-SURGICAL TREATMENT IN PATIENTS WITH KNEE OSTEOARTHRITIS - A RANDOMIZED CONTROLLED TRIAL	unclear exercise principles

226	V. S. Husby, O. A. Foss, O. S. Husby and S. B. Winther	2018	Randomized controlled trial of maximal strength training vs. standard rehabilitation following total knee arthroplasty	unclear exercise principles
227	B. Karaismailoglu	2021	High-Intensity Strength Training, Knee Pain, and Knee Joint Compressive Forces in Adults With Knee Osteoarthritis	unclear exercise principles
228	S. Y. Lee, J. H. Choi, S. R. Kim, Y. G. Park, H. J. Lee and W. Jeong	2023	Functional outcomes and length of stay with early and high-intensity rehabilitation after simultaneous bilateral total knee arthroplasty	unclear exercise principles
229	Z. F. Lin, T. S. Liu, Z. L. Hu, W. T. Que, H. M. Qiu and L. Chen	2022	Effects of Different Running Intensity on Serum Levels of IL-6 and TNF-alpha in Patients with Early Knee Osteoarthritis	unclear exercise principles
230	S. Messier, S. Mihalko, D. Beavers, B. Nicklas, P. DeVita, J. Carr, D. Hunter, K. Bennell, A. Guermazi, M. Lyles and R. Loeser	2019	Is Long-term High-intensity Strength Training Beneficial or Harmful for Knee Osteoarthritis Patients? The Strength Training and Arthritis Trial (START)	unclear exercise principles
231	J. Multanen, T. Rantalainen, H. Kautiainen, R. Ahola, T. Jamsa, M. T. Nieminen, E. Lammentausta, A. Hakkinen, I. Kiviranta and A. Heinonen	2017	Effect of progressive high-impact exercise on femoral neck structural strength in postmenopausal women with mild knee osteoarthritis: a 12-month RCT	unclear exercise principles
232	J. Scheer	2021	The Effect of high-intensity Strength Training for Knee Pain and Force occurring in the Knee Joint in Adults with Knee Osteoarthritis	unclear exercise principles
233	D. Vasileiadis, G. Drosos, G. Charitoudis, I. A. Dontas and J. Vlamis	2022	The Efficacy of High-Intensity Preoperative Physiotherapy Training on Postoperative Outcomes in Greek Patients Undergoing Total Knee Arthroplasty: A Quasi-Experimental Study	unclear exercise principles
